# Diagnosis of abdominal pregnancy still a challenge in low resource settings: a case report on advanced abdominal pregnancy at a tertiary facility in Western Kenya

**DOI:** 10.11604/pamj.2018.31.239.17766

**Published:** 2018-12-20

**Authors:** Sahara Shurie, John Ogot, Philippe Poli, Edwin Were

**Affiliations:** 1 Department of Reproductive Health, College of Health Sciences Moi University, Eldoret, Kenya

**Keywords:** Abdominal pregnancy, advanced abdominal pregnancy, ectopic pregnancy

## Abstract

Abdominal pregnancy is a rare form of ectopic pregnancy, occurring in 1: 10,000 to 1: 30,000 pregnancies and accounting for up to 1.4% of all ectopic pregnancies. It is classified as primary or secondary depending on the site of fertilization. However, when it does happen, it may remain unnoticed until term because the pregnancy can appear normal during clinical examination. Advanced abdominal pregnancy is associated with high mortality rate for both the mother and the baby at 1-20% and 40-95% respectively. We report a case of a 30-year-old female para 2+0, gravida 3 at 35^+1^ who presented at a Tertiary facility in Eldoret Kenya with one-day history of per vaginal bleeding and 2 weeks' history of no fetal movements. The importance of this case report is to highlight the challenges associated with diagnosis of advanced abdominal pregnancy in low resource settings. Ultrasound alone cannot be relied on to make the diagnosis. Whenever an induction is not working, abdominal pregnancy should be considered.

## Introduction

Abdominal pregnancy is rare form of ectopic, occurring in 1: 10,000 to 1: 30,000 pregnancies and accounting for up to 1.4% of all ectopic pregnancies. It is classified as primary or secondary depending on the site of fertilization [1]. It is frequently missed in routine antenatal care. However, when it does happen, it may remain unnoticed until term because the pregnancy can appear normal in examination. The maternal mortality rate is estimated between 0.5-18% while perinatal mortality is reported to be higher as 40-95% [2]. Most of the cases which progress to term are usually asymptomatic and the diagnosis is made after failed induction of labour or during laparotomy. For symptomatic abdominal pregnancy, studies have shown that the clinical presentation depends on the gestational age [2, 3]. Advanced abdominal pregnancy can be discovered during elective Cesarean section [4]. Few cases of fetal survival have been reported though rare [5]. Advanced abdominal pregnancy is associated with high mortality rate for both the mother and the baby at 1-20 % and 40-95% respectively [6]. There are no specific clinical signs and symptoms for abdominal pregnancy hence making diagnosis difficult [7]. Most third trimester pregnancies are secondary abdominal pregnancies. This case report helps to demonstrate the challenges of diagnosing advanced abdominal pregnancy in low resource settings. Due to its unique presentation, case reports are still important to improve diagnosis and management of advanced abdominal pregnancy.

## Patient and observation

We report a case of a 30-year-old female para 2+0, gravida 3 at 35^+1^ who presented at a Tertiary facility in Eldoret Kenya with one-day history of per vaginal bleeding and 2 weeks' history of no fetal movements. The previous pregnancies were both spontaneous vertex delivery, no complications. On abdominal examination, the fundal height was 30 weeks, cephalic presentation, No fetal heart rate. There was no tenderness on abdominal examination. Fetal parts were not easily palpable. On speculum examination, slight blood oozing from the cervix. An impression of Antepartum hemorrhage (differential diagnosis of placenta Previa) with intrauterine fetal demise was made. Formal obstetric scan done showed Intrauterine fetal demise at 27+4 weeks, low lying placenta. Patient was admitted to the antenatal ward and decision to induce with Misoprostol was made. Patient received 5 doses of misoprostol (100mcg sublingual). The 4^th^ dose of misoprostol was given together with catheter (60mls of Normal saline). Once the catheter fell, the patient was transferred to the Labour ward floor, 2cm dilated and started on Oxytocin. There was no progress with oxytocin for 48 hrs. Decision to reevaluate the patient was made. A bedside obstetric ultrasound was done which showed empty uterus, chronic uterine rupture with differential diagnosis of abdominal pregnancy was made. Patient was taken to theatre for Explorative Laparotomy.

**Figure 1:**
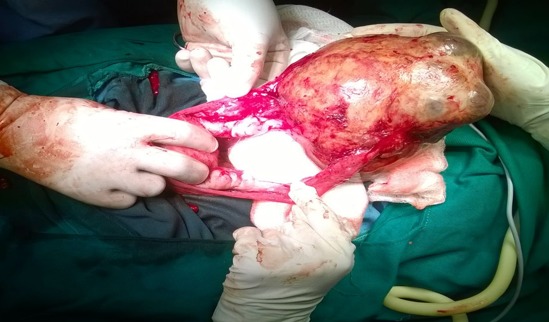
Intraoperative findings of the cystic mass with its attachments

**Figure 2:**
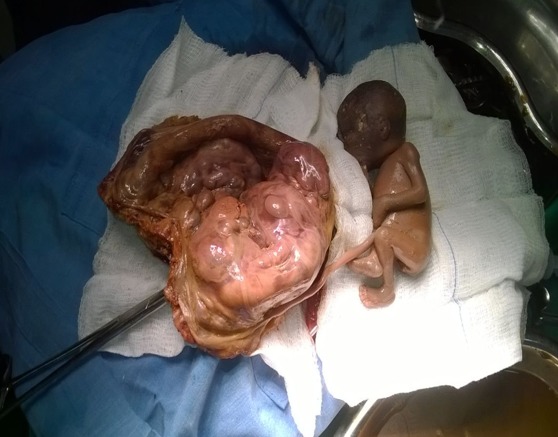
The placenta and the fetus

**Intraoperative findings:** There was a cystic mass about 20*20cm extending from the pelvis to Epigastrium, brown in colour (Figure 1, Figure 2). The Mass was attached to the anterior abdominal wall and the mesentery posteriorly. The fallopian tubes were attached on either sides of the inferolateral aspect of the mass. The ovaries were partially attached to the inferior lateral aspect of the mass bilaterally. The greater omentum was partially attached to the superior aspect of the mass. The uterus was grossly normal and free. The stomach, bowel, liver and spleen were grossly normal. The cystic mass was easily separated by blind dissection from the anterior abdominal wall, the mesentery and the greater omentum. The ovaries were easily separated from the infero-lateral aspect of the mass. Bilateral partial salpingectomy was done since both the fallopian tubes were tightly adherent to the mass.

## Discussion

The diagnosis of abdominal pregnancy is often missed during antenatal care [7, 8]. In this case ultrasound showed intrauterine pregnancy [2]. Most patients with abdominal pregnancy have persistent abdominal or gastrointestinal symptoms during pregnancy [9]. Abdominal pregnancy should be suspected when body parts are easily palpated on clinical exam [8]. In our case, it was not picked during abdominal examination. This may be due to lack of consistency in examination of the patient. Our patient was on follow up at a peripheral facility which did not have access to imaging and it was not suspected on clinical examination. The symptomatology of advanced abdominal pregnancy is vague hence the difficulty in diagnosing in resource limited settings. Magnetic Resonance Imaging is the most accepted method of diagnosing abdominal pregnancy. Ultrasound is suitable for screening abdominal pregnancies [10]. Based on the formal ultrasound from the radiology department, it was an intrauterine fetal demise and at that point induction of labour was the best option of managing the patient. After unsuccessful induction, other differential diagnosis were entertained and a bedside ultrasound was done at the labour floor which showed empty uterus. Chronic uterine rupture and advanced abdominal pregnancy were considered and the patient was taken to theatre for Laparotomy. The diagnostic criteria for abdominal pregnancy, treatment timing, operative consideration, postoperative follow-up deserve our attention. Obstetricians need to be aware on how to diagnose abdominal pregnancy early and minimize the risks and complications to the patients [7]. The diagnosis of abdominal pregnancy requires high index of suspicion. The clinical presentation of abdominal pregnancy is atypical. Where abdominal pregnancy is suspected, other clinical features like abdominal tenderness, palpable fetal parts should be looked for. In our case, the typical clinical features of abdominal pregnancy were absent.

MRI is the best modality for confirmation of abdominal pregnancy. In cases where abdominal pregnancy is suspected, MRI should be done. Management of placenta in advanced abdominal pregnancy is a contentious issue. Incomplete removal of the placenta may result torrential hemorrhage due to lack of uterine contraction which is absent in abdominal pregnancy. Complete removal of placenta is done only when the blood supply can be identified and meticulous ligation can be done [11]. Therefore, placenta should be removed if it is safe and the patient followed up for possible complication [12]. There is role for methotrexate together with Leucovarin to aid placental autolysis in cases where removal of the placenta is detrimental to the patient. In our case, the placenta was confined within the cystic mass, hence no difficulty in removal. The main treatment option of advanced abdominal pregnancies is open surgery [7]. As we have seen from literature, it can be missed easily and MRI is the better option where advanced abdominal pregnancy is suspected [12-14]. MRI is able to give the details of the abdominal pregnancy including the location of the placenta which helps in deciding the treatment options for the patient [8]. In advanced abdominal pregnancy, the placenta is located near the uterine wall where there is a lot of blood supply. This explains the long duration of fetal survival almost to term. There is fetal growth retardation associated with advanced abdominal pregnancy but there is no increase in fetal malformation reported. This case is presented to highlight the dilemma associated with the diagnosis and management of abdominal pregnancy in low resource settings.

## Conclusion

The importance of this case report is to highlight the challenges associated with diagnosis of advanced abdominal pregnancy in low resource settings. Ultrasound alone cannot be relied on to make the diagnosis. There is need to consider MRI where advanced abdominal pregnancy is suspected. Whenever there is failed induction of labour, advanced abdominal pregnancy is a possibility.

## Competing interests

The authors declare no competing interest.
